# The declining antibody level of measles virus in China population, 2009–2015

**DOI:** 10.1186/s12889-018-5759-0

**Published:** 2018-07-24

**Authors:** Ying Hu, Peishan Lu, Xiuying Deng, Hongxiong Guo, Minghao Zhou

**Affiliations:** The Department of Expanded Program on Immunization, Jiangsu Provincial Center for Disease Prevention and Control, 172 Jiangsu Road, Nanjing, 210009 China

**Keywords:** Measles virus, Antibody level, Herd immunity, Protection rate of population, Measles elimination

## Abstract

**Background:**

To eliminate measles, the coverage of more than 90% vaccine is required in China. Nonetheless, the measles incidence still reached to 3.88 per 100,000 in 2014, which is far more than the target of 1 per 1,000,000. Moreover, there is little national surveillance to measles antibody level indicating herd immunity status in China.

**Methods:**

We detected the level of antibody to measles using commercially available indirect enzyme-linked immunosorbent IgG assays, and calculated the protection rate of population (PRP) to measles virus infection among health peoples in China.

**Results:**

During the years 2009–2015, among the Chinese population aged 0–56, PRP was 94.7, 91.6, 91.6, 84.2, 82.1, 81.0, 75.4%, respectively. Among all age bands, the PRP is lowest among children less than 12-month-age, followed by people over 15 years old.

**Conclusion:**

Measles antibody level among healthy population has been declined since 2012, supplemented measles vaccination activity may be necessary to eliminate measles in China.

## Background

Measles is a highly contagious and vaccine-preventable illness. However, measles has been eradicated in the Region of the Americas due to the safe and effective vaccine, and the other five regions of World Health Organization (WHO) have adopted measles elimination goals and are trying to eliminate measles by increasing measles-containing vaccine coverage [1, 2]. In China, a two-dose schedule of measles vaccine was introduced in 1986, and vaccination age modified in 2005 [3]. Up to now, the coverage of measles vaccine hit a high level of over 90% since 2006. However, the number of measles cases reported by China reached around 100,000 during 2005–2008, responsible for over 90% of cases in the Western Pacific Region [4–6].

In order to achieve the measles elimination goal, 27 of the 31 mainland provinces of China conducted unsynchronized province-wide supplementary immunization activities against measles between 2003 and 2009, and a nationwide measles supplementary immunization activity was conducted in September 2010, which meant that the reported measles cases dropped dramatically to 0.76 per 100,000 in 2011 from 2.8 per 100,000 in 2010 [7, 8]. At the same time, school entry immunization checks and plans for reaching hard-to-reach communities were implemented. Nonetheless, the measles incidence still reached to 3.88 per 100,000 in 2014, which is far more than 1 per 1,000,000 [5]. Therefore, there is still a serious challenge to overcome on the way of eliminating measles.

Mathematical models have estimated that the herd immunity threshold necessary to interrupt measles transmission in the United States is 93–95%; below this level a measles outbreak could be sustained if the virus was introduced [9, 10]. In China, there is little national antibody level of measles virus. To investigate the herd immunity level of measles antibody, we monitored measles antibody among healthy people living in Jiangsu province, located the eastern part China, from 2009 to 2015.

## Methods

### Subject and sample collection

Participants were aged from 1 month to 54 years old. The participants were classified into four groups: 0~ 12 months, 1~ 4 years,5~ 14 years, over 15 years. For those less than 18 years old, a written consent form was obtained their parents before collecting blood samples. The 2 ml of whole blood sample was collected into 5 ml vacuumed tube with EDTA-K3, and then centrifuged at 3000 rpm for 15 min to isolate serum. The serum specimens were transferred to Jiangsu Provincial Center for Disease Control and Prevention Measles\Rubella Net Laboratory to measure IgG concentration. From 2009 to 2015, a total of 18,100 healthy subjects were enrolled in immunity level surveillance of measles virus.

### Laboratory testing

Commercially available indirect enzyme-linked immunosorbent IgG assays (Serion ELISA classic for measles virus IgG) was used for the detection and qualitative determination of IgG antibody to measles virus. The concentration of IgG antibody to measles virus was calculated according to the lot-specific 4-parameter logistic standard curve provided by Institute Virion/Serion GmbH for individual Serion ELISA classic test. Four parameters were: lower asymptote (OD), slope of the curve, inflection point, and upper asymptote, which were indicated as A, B, C, and D, respectively. All sera specimens were tested at a single dilution of 1:400. Samples were considered as having protective ability if the concentration was ≥200, and no protective ability if˂200. The protection rate of population (PRP) is calculated as the formula: PRP = the number of the specimens having protective ability/the number of no protective ability*100%.

### Statistical analysis

Pearson’s Chi-square test was used to compare the protection rate of population between various years; if the expected frequency was less than five, then Fisher’s test was used instead. Statistical significance was defined by a two-sided *p*-value of less than 0.5. Chi-square test were performed using OpenEpi software version 2.3.1 online (http://www.openepi.com/Menu/OE_Menu.htm).

## Result

A total of 18,100 serum specimens were tested for measles IgG concentration (IU/mL) among 2009–2015. The percent of participants varied by age (less than 1 year, 10.8%; 1–4 years, 20.5%; 5–14 years, 30.3%; 15–56 years, 39.0%), sex (male, 51.7%; female,48.3%).

**Fig. 1:**
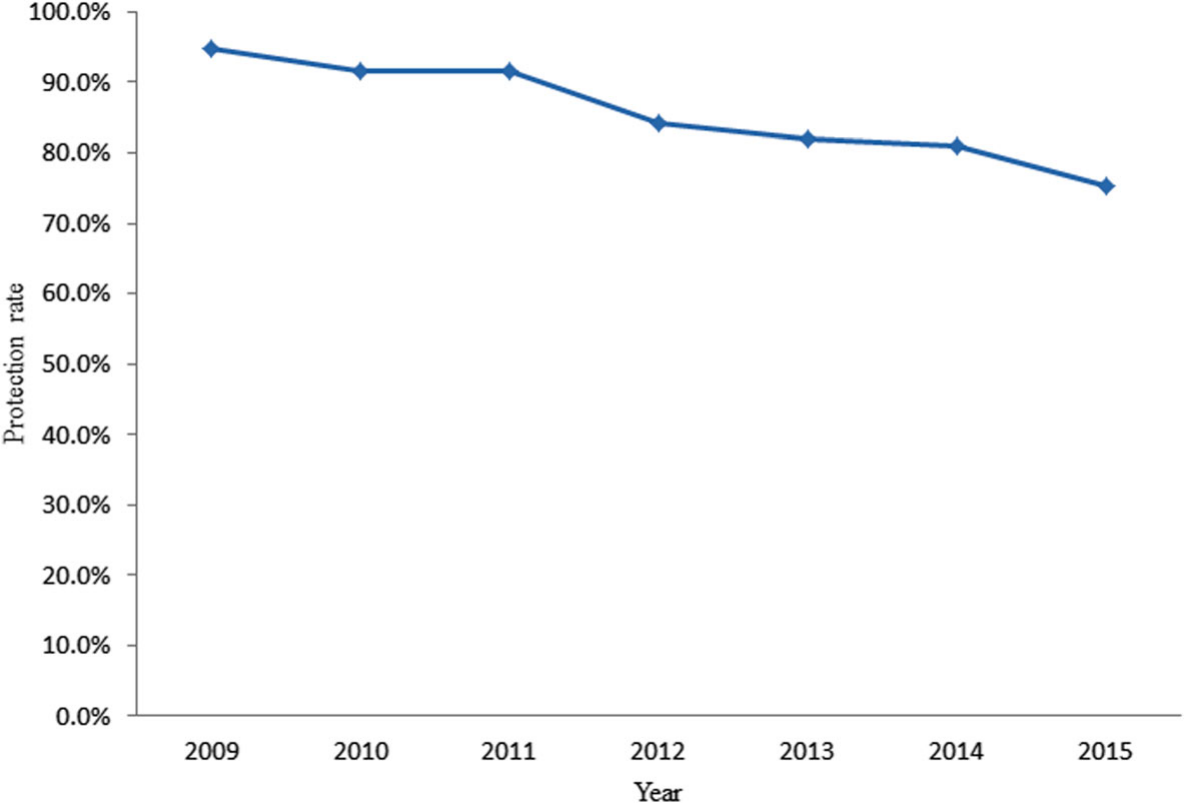
the protection rate of measles virus among health population in China, 2009 to 2015

**Fig. 2:**
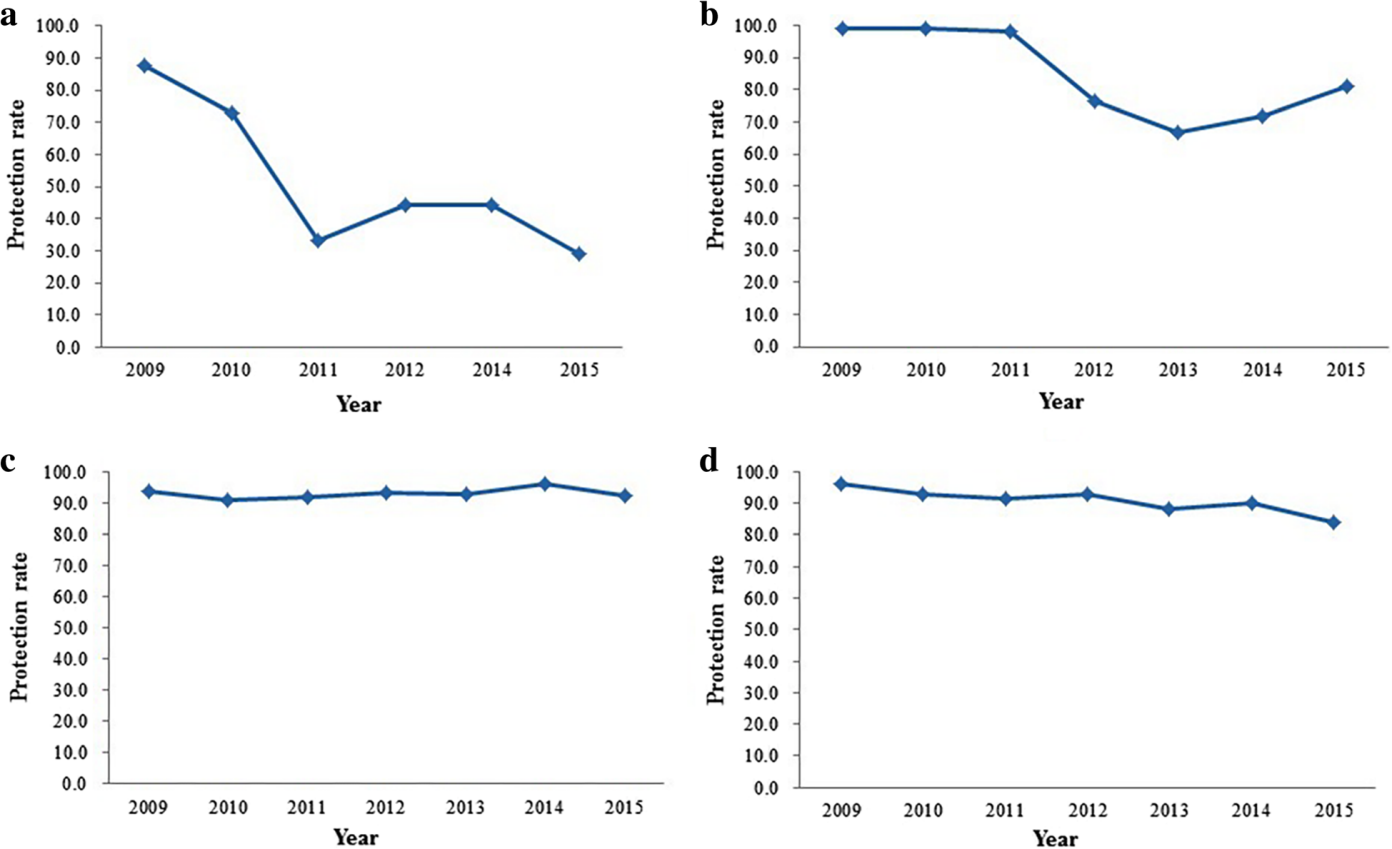
the age specific protection rate of measles virus among health population in China, 2009 to 2015. **a**: the protection rate of measles virus among children less than 12 months; **b**, the protection rate of measles virus among children between one-year-old and four-year-old; **c**, the protection rate of measles virus among children between five-year-old and 14year-old; **d**, the protection rate of measles virus among population more than 14 years old

During the years 2009–2015, among the Chinese population aged 0–56, PRP was 94.7, 91.6, 91.6, 84.2, 82.1, 81.0, 75.4%, respectively. As shown in Fig. 1, the PRP has been declined from 2009 to 2015. From 2009 to 2012, 2014 and 2015, PRP among less than 12 month age children was 87.5, 72.7,33.3,44.3,29.2%, respectively. PRP reduced rapidly since 2012. During 2009–2015, among one to 4 years old children, PRP was 98.9, 99.1, 98.0, 76.6, 66.7, 71.8, 81.0%, respectively (Fig. 2b). PRPs were higher among 2009–2011, then decreased rapidly to 66.7% in 2013 and maintained the level less than 81.0%. Among the five to 14 years old population, PRP was 93.9, 91.0, 92.1, 93.2, 93.1, 96.5, and 92.6% from 2009 to 2015, respectively (Fig. 2c), which are highest values among the participants of all age bands. Among 15 to 56 years old population, PRP was 96.2, 92.8, 91.5, 92.6, 88.8, 90.0, 83.7%, respectively (Fig. 2d). It also maintained a higher level other than in 2015.

As shown in Fig. 2, within any 1 year among 2009–2015, PRP is lowest among children less than one-year-old. Among 1 to 4 years old children, PRP was lower in 2012–2014 and less than 80%. PRP reached more than 91% among children aged from 5 to 14. Among people aged over 15 years old, PRP was less than 90% only in 2013 and 2015. In 2015, it was only 83.7%.

## Discussion

Our results show that overall PRP remained high for measles during 2009–2011. However, it has been declining since 2012. In order to confirm the reason for the decrease of PRP, we analyzed the percent of less than one-year-old participants among all participants in various years. As we knew, the first dose of measles vaccine was given about 8 months old. So the lower PRP will appear if more less than 12 months old children were enrolled. The percent of less than one-year-old participants exceeded 10% since 2012, and reached to 22.1% in 2015. Nonetheless, it contributed to the decreasing PRP partially. For example, PRP is higher in 2012 than in 2014, although the percent of children less than 8 months old is higher in 2012 than 2014. Furthermore, the PRP of 1–4 years old during 2009–2011 was higher than that during 2012–2015, and the PRP of over 15 years old in 2015 was only 83.7%. This indicates that PRP really decreased in recent years, and implies that the number of susceptible people to measles may have already increased. Previous study showed that 93–95% PRP is required to interrupt a measles outbreak. However, our results reveal that PRP is far from this level in China since 2012. In 2013, measles epidemic resurgence occurred in most of the provinces in China [5, 6, 11, 12]. Less than 1 year old children and adults over 15 years old are the two major populations with measles epidemic [12–14]. In our study, these two populations have lower PRP than other two age bands populations. Between 2009 and 2010, Jiangsu province conducted supplemented measles vaccination activity which may contribute to decrease measles outbreak and improve higher PRP during 2009–2011. In addition, although measles vaccination has been required to cover more than 95% children in Jiangsu province, more than 93% immunity screen against measles virus remains unformed. There are still many populations susceptible to measles. Previous practice confirmed that the supplemented measles vaccination activity can significantly decrease the incidence of measles [3, 15]. For this reason, it may be more important to implement supplemented measles vaccination activity targeting the susceptible population than general population.

There are some populations who remain susceptible to measles as they received neither routine immunization nor the supplementary immunization, and the accumulation of such populations could also cause the outbreaks in the country.

## Conclusion

The PRP to measles virus has been declined since 2009 in China, this phenomenon was observed in all age-band children other than 5–14 age-band, and maybe partly responsible for continued measles outbreak in China. Therefore, periodical supplement immunization is necessary to achieve measles elimination earlier in China.

## Abbreviation

PRP: The protection rate of population
